# Long‐Term Follow‐Up of Inducible Bundle Branch Reentrant Ventricular Tachycardia in Patients Without Structural Heart Disease: Beyond the Electrophysiology Laboratory

**DOI:** 10.1111/jce.16777

**Published:** 2025-07-01

**Authors:** Muhieddine Omar Chokr, Haroldo Heitor Ribeiro Filho, Roberto Alvarez Coello, Rodrigo Melo Kulchetscki, Lucas Moura, Francisco Darrieux, Denise T. Hachul, Luciana Sacilotto, Tan C. Wu, Pedro M. Vandoni, Italo B. Souza, Pedro Linhares, Cristiano F. Pisani, Carina A. Hardy, Mauricio I. Scanavacca

**Affiliations:** ^1^ Arrhythmia Unit, Heart Institute University of Sao Paulo Medical School são Paulo Brazil Brazil

## Abstract

**Background:**

Bundle branch reentrant ventricular tachycardia (BBR‐VT) is a rare form of VT occurring in patients with structural heart disease (SHD). Rarely, it can also occur in the absence of SHD. Understanding its clinical and electrophysiologic (EP) properties and outcomes post‐catheter ablation (CA) is crucial.

**Objective:**

We present a series of patients with bundle branch reentrant ventricular tachycardia (BBR‐VT) and structurally normal hearts, comparing their clinical and electrophysiological characteristics, as well as long‐term outcomes, with those of patients with the classic form of BBR‐VT, in whom the arrhythmia was induced in the presence of ventricular dysfunction.

**Methods:**

All cases of BBR‐VT diagnosed during electrophysiological (EP) studies in our lab were evaluated. Clinical characteristics and EP findings were described.

**Results:**

Sixteen patients (12/16 male; mean age 50 ± 21 years) with BBR‐VT were studied from 2009 to 2020, with a mean follow‐up of 70 ± 16 months. Notably, nearly half (7/16; 43%) had no structural heart disease (SHD). Among these, three had myotonic dystrophy, two had SCN5A mutations, one had ajmaline‐induced BBR‐VT, and one had idiopathic BBR‐VT. BBR‐VT was induced with a mean cycle length of 322 ± 22 ms and was well tolerated in 10/16 patients. Right bundle branch catheter ablation was performed using an 8 mm solid‐tip catheter, leading to an HV interval increase from 72 ± 9 ms to 100 ± 23 ms. One patient developed total AV block. Postprocedure, device implantation was required in 13/16 patients (dual‐chamber pacemaker: 10; ICD: 3). Notably, no patient experienced VT recurrence during follow‐up.

**Conclusion:**

BBR‐VT is traditionally associated with dilated cardiomyopathy, but it can also occur in structurally normal hearts. Myotonic dystrophy and SCN5A mutations may underlie some cases. Right bundle branch ablation is an effective treatment, though device implantation is usually required.

## 1 Introduction

Bundle branch reentrant ventricular tachycardia (BBR‐VT) is a rare form of arrhythmia, accounting for a small proportion of ventricular tachycardia (VT) cases [1, 2]. It is predominantly observed in patients with dilated cardiomyopathy and associated atrioventricular (AV) conduction abnormalities. The arrhythmia is characterized by a macro‐reentrant circuit involving the left and right bundle branches (LB and RB) and is a recognized cause of syncope and sudden cardiac death (SCD) in this population.

Although BBR‐VT is well‐documented in the context of structural heart disease (SHD), its incidence in patients without underlying SHD remains poorly defined. Emerging reports have identified BBR‐VT in patients with muscular dystrophy [3, 4], SCN5A mutations [5], and even in individuals without comorbidities but with distinct or functional His‐Purkinje conduction abnormalities [6, 7].

In this study, we present a case series of patients with inducible BBR‐VT, highlighting a significant prevalence of structurally normal hearts in this cohort. The analysis provides a comprehensive description of the clinical and electrophysiological characteristics of these cases, highlighting the differences in comparison to the population with structural heart disease and proposes a systematic approach for the evaluation and management of patients presenting with BBR‐VT in the electrophysiology (EP) laboratory.

## 2 Objective

We present a series of patients with bundle branch reentrant ventricular tachycardia (BBR‐VT) and structurally normal hearts, comparing their clinical and electrophysiological characteristics, as well as long‐term outcomes, with those of patients with the classic form of BBR‐VT, in whom the arrhythmia was induced in the presence of ventricular dysfunction.

## 3 Methods

A retrospective analysis was conducted on patients identified through our institution's ventricular tachycardia (VT) ablation database. All individuals with a confirmed diagnosis of inducible bundle branch reentrant ventricular tachycardia (BBR‐VT) who underwent evaluation and treatment between 2009 and 2020 were included. Written informed consent was obtained from all participants before the electrophysiological procedure, in accordance with institutional and ethical guidelines.

### 3.1 Electrophysiology Procedure

All procedures were performed in the dedicated electrophysiology laboratory of the Heart Institute at the University of São Paulo Medical School. General anesthesia was predominantly administered using inhalational agents such as sevoflurane, complemented by propofol and/or intravenous fentanyl as needed. In patients with structural heart disease (SHD), additional monitoring measures were employed, including the placement of a central venous catheter, arterial line, and urinary catheter for accurate assessment of urinary output.

A standardized programmed ventricular stimulation (PVS) protocol was employed, with driving cycle lengths of 600 ms and 430 ms, incorporating up to three extrastimuli at a minimum coupling interval of 200 ms. Stimulation was delivered at two predefined sites in the right ventricle (apex and outflow tract) using EP‐TRACER software version 2.2 (Cardiotek B.V., Netherlands). Electroanatomical mapping was not available in any of the patients. In 2 of the 16 patients, a decapolar catheter was advanced to the left septum via a retrograde aortic approach, allowing documentation of retrograde activation of the left bundle branch (LB).

Ventricular tachycardia (VT) ablation was performed using an 8‐mm solid‐tip catheter (St. Jude Medical Inc., St. Paul, MN, USA), operating at a power of 50 W with a maximum temperature setting of 55°C. VT mapping and ablation were localized to the right bundle (RB) upon confirmation of a diagnosis of bundle branch reentrant ventricular tachycardia (BBR‐VT).

The success of the procedure was evaluated at its conclusion by confirming the presence of right bundle branch block (RBBB) and repeating a postablation PVS protocol at a minimum of one site within the right ventricle. Procedural outcomes were classified into three categories:

- **Complete success**: No BBR‐VT was inducible following ablation.
- **Partial success**: Baseline clinical BBR‐VT was no longer inducible, though at least one nonclinical, faster VT was induced.
- **Unsuccessful**: Clinical BBR‐VT remained inducible postablation.

### 3.2 Definition of BBR‐VT

**Figure 1:**
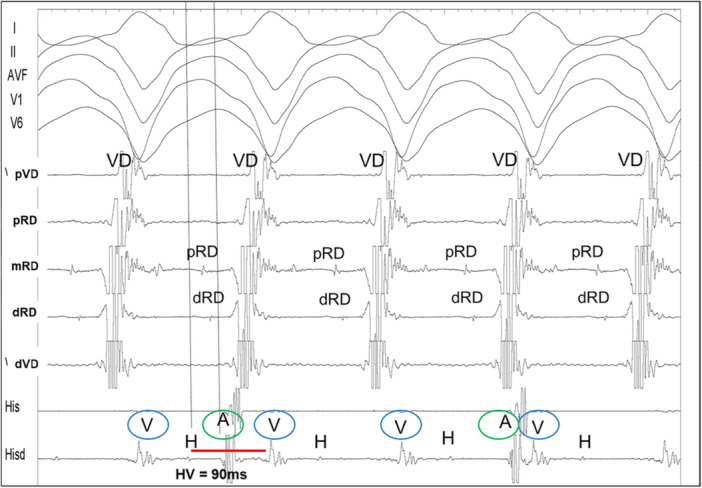
Intracavitary signals of a BBR‐VT. Note a positive HV interval that was longer than in sinus rhythm (90 ms). Also, in the RV catheter, one can see the proximal (pRB) and distal (dRB) right bundle signals in a proximal‐to‐distal fashion, both of them located about 20 ms after the His potential (H). Dissociated atrial signals can also be identified (A).

**Figure 2:**
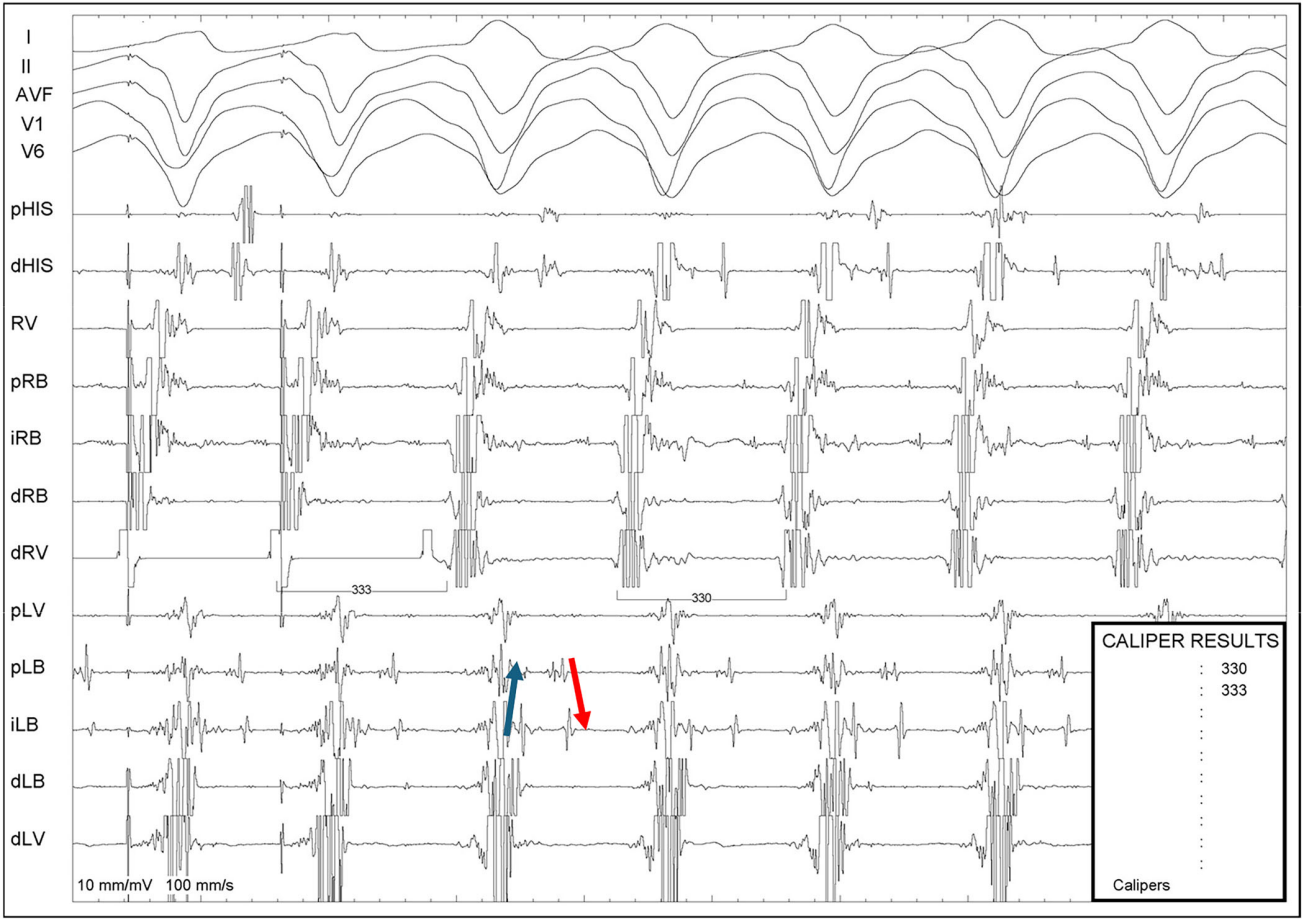
Post pacing interval (PPI) minus tachycardia cycle length (TCL) less than 30 ms, with an increase in the HV interval compared to sinus rhythm, thus confirming the diagnosis of bundle branch reentrant ventricular tachycardia. The catheter positioned on the left side likely records retrograde activation of the posterior fascicle (blue arrow), while the (red arrow) possibly represents antegrade activation of the antero superior fascicle, which is passively activated.

The diagnosis of BBR‐VT was performed using the following criteria: (A) Changes in the H‐H (RB‐RB or LB‐LB) interval during VT preceded changes in the VV interval; (B) His or bundle branch potential preceding each ventricular activation, with H‐V interval of tachycardia equal or greater than baseline HV (Figure 1); (C) Post pacing interval (PPI) minus tachycardia cycle length (TCL) less than 30 ms at the apex of the right ventricle (Figure 2); (D) V‐H interval prolongation at the beginning of the tachycardia induction; (E) Inability to induce VT after right bundle branch ablation.

### 3.3 Follow‐Up

Patients were followed for 30 days postprocedure and subsequently every 3 months, or earlier if clinically indicated. Clinical characteristics and procedural data were collected from medical records and through telephone contact when necessary.

### 3.4 Statistical Analysis

Continuous variables with normal distribution are presented as mean ± standard deviation (SD), while skewed distributions are reported as median with interquartile range (IQR). Categorical data are expressed as counts and percentages. The normality of the distributions was assessed using the Kolmogorov‐Smirnov test. All statistical analyses were performed using SPSS Statistics® software, version 21.0 (IBM Corp., Armonk, NY, USA).

## 4 Results

A total of 16 patients diagnosed with BBR‐VT were studied between 2009 and 2020. The mean age was 50 ± 21 years, and 75% of the cohort were male. The mean ejection fraction was 43 ± 18%. Intraventricular conduction disturbances on surface electrocardiogram (ECG) were observed in all but three patients. Notably, nearly half of the cohort (7/16; 43%) exhibited a normal left ventricular ejection fraction (Table 1). Among those with cardiomyopathy (9/16; 57%), the predominant etiology was idiopathic (4/9), followed by ischemic (3/9), sarcoidosis (1/9), and hypertensive (1/9). Among the subgroup with structurally normal hearts (7 patients; 43% of the cohort), 3/7 were diagnosed with myotonic dystrophy, 2/7 with SCN5A mutations (both presenting with atrial flutter years before the ventricular arrhythmia), 1/7 following ajmaline administration, and 1/7 remained of undefined etiology. The most frequent clinical presentations included palpitations, chest pain, and syncope. The primary indication for the electrophysiology procedure was unexplained syncope in 5 patients and documented tachycardia in the remaining cases. All patients with syncope, for whom the procedure was indicated, presented with ventricular dysfunction. Tachycardia was poorly tolerated in six patients (Table 1). Fibrosis was identified in 23% of the patients who underwent MRI (4/13), all in the group with ventricular dysfunction. Only one patient had a pre‐existing implanted electronic device whose indication was for primary prophylaxis. The mean clinical follow‐up duration was 70 ± 16 months.

**Table 1:** Clinical characteristics of all patients during follow‐up, the first seven refer to patients with structurally normal hearts.

Number/Gender	Age	Cardiopathy	Clinical manifestations	Other conditions	Ejection fraction	Antiarrhythmics	Delayed myocardial enhancement on MRI
1. Male	18	None	Palpitations	None	68%	Propafenone + Propranolol	No MRI
2. Female	37	None	Palpitations + thoracic pain	None	67%	Amiodarone + Atenolol	No MRI
3. Male	45	None	Palpitations	Atrial flutter + AF + AVNRT	66%	Amiodarone	None
4. Female	23	None	Palpitations + thoracic pain	Steinert Dystrophy	55%	Atenolol	None
5. Male	38	None	Palpitations	Steinert Dystrophy	67%	Atenolol	None
6. Female	37	None	Ventricular tachycardia during effort	SCN5A positive, Hypertension, and Hypothyroidism	55%	None	None
7. Male	27	None	Palpitations + thoracic pain during effort	SCN5A positive	66%	Amiodarone	None
8. Male	43	Sarcoidosis	Poorly tolerated palpitations	CKD + Pulmonary Sarcoidosis	47%	Amiodarone	No MRI
9. Male	77	Ischemic	Palpitations	MI + CABG + AF	35%	Amiodarone + Carvedilol	Yes
10. Female	64	Idiopathic	Syncope	AF + Hypothyroidism	21%	Amiodarone + Carvedilol	None
11. Male	67	Ischemic	Syncope + palpitations	Ashtma + Hypertension + MI + CABG + CKD	20%	Amiodarone + Carvedilol	Yes
12. Male	39	Idiopathic	Syncope + dispnea	Diabetes + Ashtma + PTA	25%	Carvedilol	None
13. Male	74	Ischemic	Syncope	Hypertension + MI + Coronary PTA	30%	Carvedilol	Yes
14. Male	80	Hypertensive	Syncope	Hypertension + Upper Extremity DVT	44%	Amiodarone + Carvedilol	None
15. Male	20	Idiopathic	ICD Therapy	ICD (primary prevention)	25%	Amiodarone + Carvedilol	None
16. Male	59	Idiopathic	Syncope	Coronary PTA + former smoker	35%	Amiodarone + Carvedilol	Yes

Abbreviations: Characteristics of each patient: AF, atrial fibrillation; AVNRT, atrioventricular nodal reentrant tachycardia; CABG, coronary artery bypass graft; CKD, chronic kidney disease; ICD, implantable cardiac defibrillator; MI, myocardial infarction; MRI, magnetic resonance imaging; PTA, percutaneous transluminal angioplasty.

Only two patients had a history of prior electrophysiological study both for typical atrial flutter ablation. For all other patients, the procedure was the index study.

**Figure 3:**
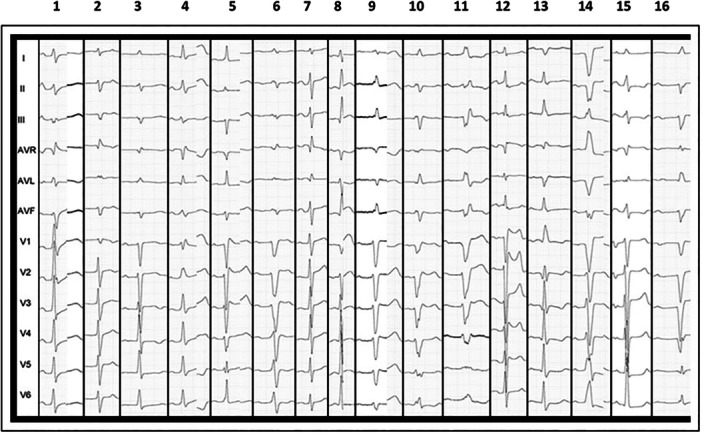
Sinus Rhythm of each patient at the beginning of the electrophysiologic study. The first seven columns correspond to patients with structurally normal hearts, while the remaining nine represent those with structural heart disease.

**Figure 4:**
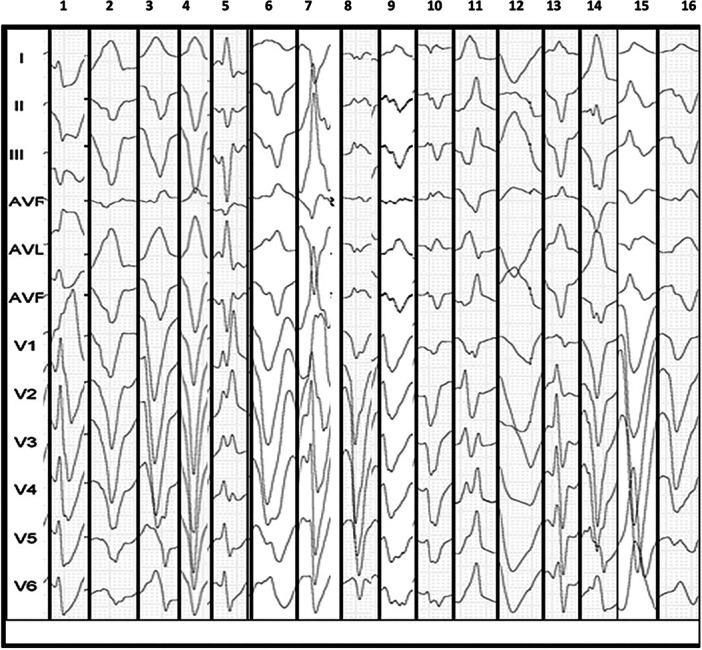
Morphologies of ventricular tachycardia in each case. The first seven columns correspond to patients with structurally normal hearts, while the remaining nine represent those with structural heart disease. Notably, a right bundle branch block morphology was observed exclusively in patients with preserved ventricular function, suggesting the left bundle as the anterograde limb of the circuit.

At the beginning of the electrophysiological study, all patients were in sinus rhythm. First‐degree atrioventricular (AV) block was identified in seven cases (Five patients in the structurally normal heart group (*p* = 0.0017), left bundle branch block (LBBB) in five cases, right bundle branch block (RBBB) in three cases, and incomplete bundle branch block in three cases (Figure 3). The induced BBR‐VT exhibited a mean cycle length of 322 ± 22 ms and was well tolerated in most patients (10/16), in patients with preserved function, the mean tachycardia cycle length was 244 ± 14 ms, while in those with dysfunction, it was 340 ± 11 ms (*p* < 0.001). Thirteen patients (81%) demonstrated the right bundle branch as the anterograde limb of the reentrant circuit, characterized by counterclockwise activation. In contrast, three patients exhibited the left bundle branch as the anterograde limb with clockwise activation, resulting in a predominant right bundle branch block (RBBB) morphology on surface ECG. Interestingly, all three patients had structurally normal hearts. (Figure 4).

The mean increase in the H‐V interval during tachycardia was 30 ± 15 ms (Figure 1), and the post‐pacing interval (PPI) minus tachycardia cycle length (TCL) was 15 ± 8 ms (Figure 2). In three cases, the diagnostic maneuver was inconclusive due to tachycardia interruption. In these instances, the diagnosis was established using alternative criteria.

**Figure 5:**
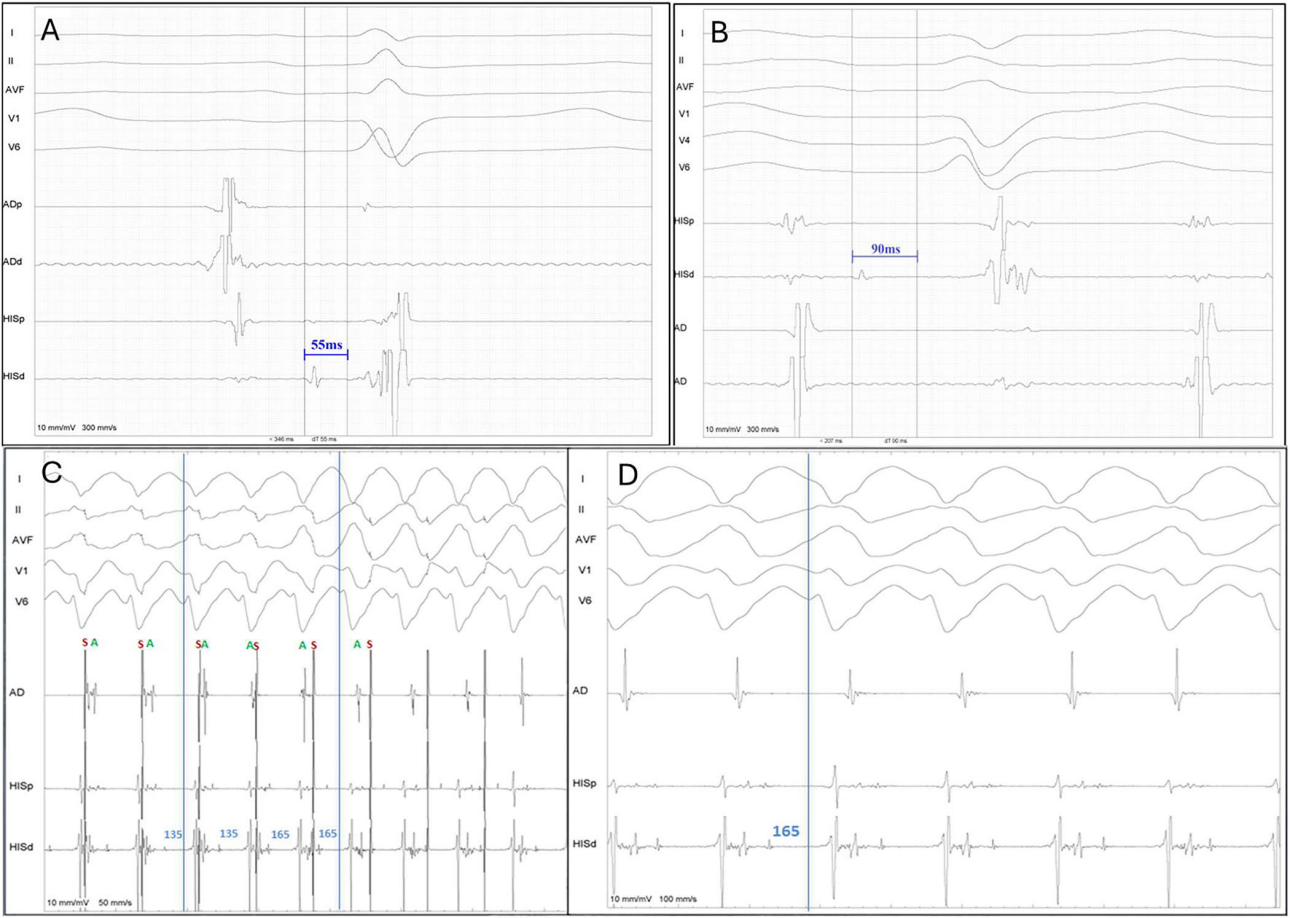
Tracings from the patient in whom BBR‐VT was induced after ajmaline induction. A: normal HV interval at baseline. B: prolonged HV interval after 50 mg of Ajmaline infusion. C: BBR‐VT induction during atrial stimulation. D: BBR‐VT with a longer HV interval during tachycardia.

In one case, a patient with a structurally normal heart and left bundle branch block (LBB) underwent an electrophysiological (EP) study for syncope evaluation, aiming to assess His‐Purkinje conduction abnormalities. Given the presence of LBB and the clinical suspicion of conduction system disease, ajmaline was administered at a dose of 1 mg/kg to unmask potential His‐Purkinje conduction delay. This resulted in a prolongation of the H‐V interval from 55 to 90 ms, suggesting a progressive conduction system impairment. During the assessment of the AV Wenckebach point, bundle branch reentrant ventricular tachycardia (BBR‐VT) was induced. As a therapeutic strategy, a pacemaker was implanted, but right bundle branch (RBB) ablation was not performed. The patient remained asymptomatic for 44 months postprocedure, with no recurrence of syncope or documented arrhythmias (Figure 5).

Right bundle branch ablation was performed in all patients, except for the one in whom BBR‐VT was induced after ajmaline infusion. Initially, the ablation catheter was positioned distally in the right septum and retracted until the right bundle branch potential was recorded, confirmed by the absence of the atrial electrogram and its occurrence 20 ms after the His potential. At this site, 2 ± 1 radiofrequency (RF) pulses were delivered, inducing RBBB after 8 ± 5 s. Extensive testing was performed, and bundle branch reentrant ventricular tachycardia was no longer inducible, with complete success in eliminating BBR‐VT in all patients who underwent right bundle branch ablation. In one patient with ischemic cardiomyopathy, another myocardial‐origin ventricular tachycardia was induced after right bundle branch ablation, requiring electrical cardioversion. The reason for not performing right bundle branch ablation in the patient in whom tachycardia was induced only after ajmaline infusion was that the infrahisian conduction disturbance was purely functional and drug‐induced. Therefore, clinical observation alone was chosen as the management strategy.

Following RBB ablation, the H‐V interval increased from 72 ± 9 ms to 100 ± 23 ms. Only one patient (with baseline QRS of 150 ms and LBBB) developed total AV block postablation. In two patients, postablation H‐V intervals were measured at 72 and 90 ms, and both remained asymptomatic during follow‐ups of 3 and 5 years without a pacemaker, respectively. Pacemaker implantation was performed in 10 patients, and three patients received immediate implantable cardioverter‐defibrillators (ICDs) during the same hospitalization.

At a mean follow‐up of 70 ± 16 months, two patients died—one due to heart failure and another due to urinary sepsis, both patients with ventricular dysfunction. Importantly, no recurrences of tachycardia were observed during the follow‐up period.

Tables 2 and 3 summarize the main differences observed in electrophysiological and clinical characteristics between patients with structurally normal hearts and those with structural heart disease. Patients with structurally normal hearts exhibited a longer baseline HV interval, greater prolongation of the HV interval during tachycardia, a longer PR interval, and a shorter QRS duration in sinus rhythm. Additionally, the rate of tachycardia induced in the electrophysiology laboratory was faster compared to that observed in patients with associated structural heart disease.

**Table 2:** Electrophysiological characteristics in patients with structurally normal hearts and with structural heart disease.

Electrocardiographic and electrophysiologic parameters	Patients with structurally normal hearts (*N* = 7)	Patients with structural heart disease (*N* = 9)	*p* value
Basal HV interval	79 ± 8 ms	71 ± 8 ms	0,.0385
Final HV interval	88 ± 6 ms	93 ± 9 ms	(NS)
HV during VT	114 ± 14 ms	102 ± 11 ms	0.0261
Basal PR interval	202 ± 9 ms	180 ± 12 ms	0.0017
QRS width in sinus	136 ± 7 ms	168 ± 13 ms	0.00003
QRS width during VT	172 ± 6 ms	181 ± 9 ms	(NS)
VT cycle length	244 ± 14 ms	340 ± 11 ms	0.0012
LBBB morphology during VT	4	9	(NS)
RBBB morphology during VT	3	0	(NS)

Abbreviations: Electrophysiological characteristics: LBBB, left bundle branch block; RBBB, right bundle branch block; VT, ventricular tachycardia.

**Table 3:** Global clinical characteristics of patients with structurally normal hearts and with structural heart disease.

Clinical Characteristics	Patients with structurally normal hearts (*N* = 7)	Patients with structural heart disease (*N* = 9)	*p* value
Age (years)	32 + −9	58 + −20	0.0048
Males	04	08	(NS)
Syncope	1	05	(NS)
History of atrial fibrillation/flutter	02	0	(NS)
History of valve replacement	0	0	(NS)
LVEF (2‐dimensional echocardiography)	63 + −5,8	31 + −9	0,0001
LVEDD (2‐dimensional echocardiography)	5,1 + −0,6	6,1 + −1,1	0.037
Late Gadolinium Enhancement	0	04	(NS)
Amiodarone before procedure	03	07	(NS)
Beta‐blockers before procedure	04	08	(NS)
Deaths during follow‐up	0	02	(NS)
Mean number of VTs	1	1,1 + −0,3	(NS)
% Need for Pacing at Final Follow‐Up	5	9	(NS)
Duration of follow‐up (months)	66 + −9	73 + −11	(NS)

Abbreviations: Global clinical characteristics: LVEDD, left ventricular end‐diastolic diameter; LVEF, left ventricular ejection fraction; VT, ventricular tachycardia.

### 4.1 Proposed Screening Algorithm for Patients Without Structural Heart Disease

**Figure 6:**
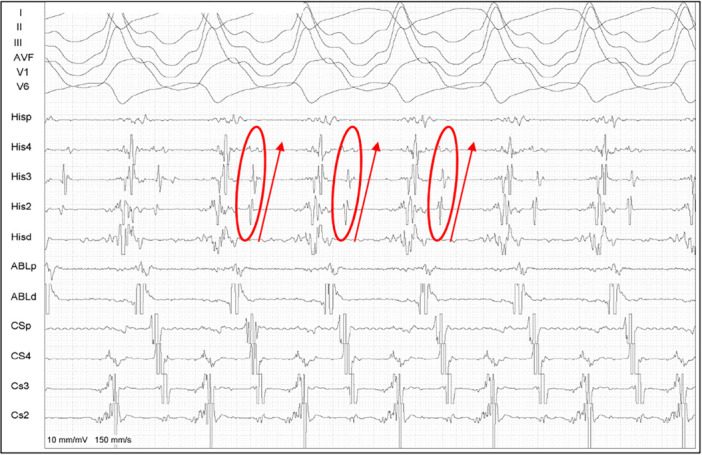
A patient with an SCN5A gene mutation and bundle branch reentrant ventricular tachycardia utilizing the left bundle branch as the anterograde limb of the circuit, presenting with right bundle branch block morphology on the electrocardiogram. This patient had undergone typical flutter ablation four years earlier. Given that the flutter was considered an isolated finding, a subsequent genetic evaluation was performed, which confirmed the presence of the genetic mutation.

In our series, some BBR‐VT diagnoses led to the identification of specific conditions. For example, in 2017, two young patients with structurally normal hearts underwent ophthalmologic evaluations following BBR‐VT diagnoses, which confirmed cataracts. Subsequent neurological and electromyoneurography evaluations revealed findings compatible with Steinert's disease. Cataracts, seen in nearly all Steinert's dystrophy patients [4, 8], underscore the importance of ophthalmologic evaluations in patients with normal hearts diagnosed with BBR‐VT. Roberts et al. [5] highlighted a possible correlation between SCN5A gene mutations and BBR‐VT in patients without structural heart disease. In our cohort, genetic investigations triggered by BBR‐VT diagnoses revealed SCN5A mutations in two patients, Interestingly, both patients had been previously diagnosed with typical atrial flutter and underwent successful ablation procedures 4 and 7 years before the onset of bundle branch reentrant tachycardia (Figure 6). Their H‐V intervals during flutter ablation were 66 ms and 81 ms, respectively, with no ventricular arrhythmias induced at that time.

Following our initial experiences with Steinert's disease, we performed ophthalmologic evaluations in three additional patients without structural heart disease. Two were diagnosed with cataracts and referred for neurological evaluation, while another could not be contacted.

**Figure 7:**
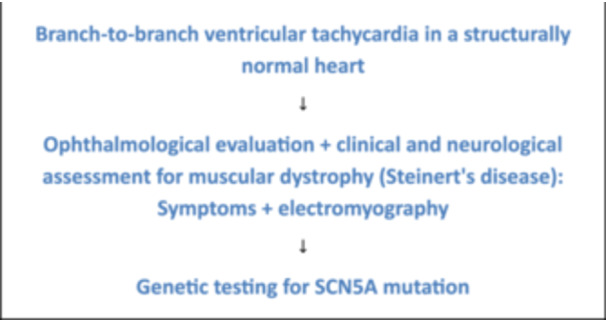
Proposed screening protocol for the diagnostic evaluation of bundle branch reentrant ventricular tachycardia in patients with structurally normal Hearts.

Based on these findings, we propose an algorithm for evaluating patients with BBR‐VT and structurally normal hearts (Figure 7), which may be useful for the electrophysiologist and the cardiologist managing the clinical follow‐up of a patient with bundle branch reentrant tachycardia induced in the electrophysiology laboratory, presenting with preserved ventricular function but, apparently, without any associated diseases.

## 5 Discussion

The BBR‐VT is a type of ventricular tachycardia typically observed in patients with significant conduction disturbances. First described by Akhtar et al. [9], it represents up to 6% of laboratory‐induced monomorphic VT cases [1, 10]. Although rare, it is often underdiagnosed and constitutes a cause of sudden death that can be effectively treated with radiofrequency ablation.

In our series, all cases induced by ventricular pacing showed an increase in the V‐H interval at the onset of tachycardia, highlighting the necessity of a critical delay in the conduction system for the arrhythmia to occur. The most common tachycardia morphology observed was left bundle branch block (LBBB), consistent with prior findings indicating that the retrograde reentrant loop preferentially occurs through the left bundle [11, 12]. This observation aligns with the fact that right ventricular stimulation, commonly used in induction, facilitates retrograde conduction through the left branch.

The treatment of BBR‐VT, first described by Langberg et al. in 1989 [13], involves RBB radiofrequency ablation, which has demonstrated a high success rate. In our series, RBB ablation rendered tachycardia non‐inducible in all patients.

All patients in our series presented with infra‐Hisian conduction disorders. Most required the implantation of an electronic device postprocedure due to the ablation‐induced RBBB, compounding pre‐existing conduction system disease. However, clinical follow‐up without pacemaker implantation was performed in two patients without structural heart disease. These patients remained asymptomatic and device‐free in the long term.

Class Ia antiarrhythmic agents are known to induce significant electrophysiological changes. In our study, the infusion of ajmaline revealed, for the first time, that acute HV interval prolongation can serve as a mechanism to enable bundle branch reentry. This novel finding raises the possibility that commonly used antiarrhythmic drugs, such as Class Ic agents, might theoretically create a substrate for bundle branch reentrant tachycardia in patients with previously undiagnosed infrahisian conduction abnormalities. In the case described, the patient remained asymptomatic for 44 months following the procedure, leading to the decision to proceed with clinical observation alone.

Interestingly, 43% of patients in our series had structurally normal hearts. Studies by Mehdirad et al. [14, 15] and Blanck et al. [10] in the 1990s similarly identified cases of BBR‐VT in patients without cardiac abnormalities, though in lower proportions. This finding suggests a potential association with underdiagnosed baseline conditions. For instance, in 2016, we reported a case of BBR‐VT in a patient with a structurally normal heart who was later diagnosed with Steinert's muscular dystrophy [16]. Approximately 32%–56% of patients with Steinert's disease present isolated conduction system defects [4, 8].

In our series, long‐term outcomes were notably favorable, with only two deaths (one of cardiac etiology) and, importantly, no mortality among patients without structural heart disease. This contrasts with prior landmark studies that underscore the inherent risks associated with BBR‐VT. Pathak et al. [17] reported a higher incidence of arrhythmia recurrence and adverse cardiac events following catheter ablation, reflecting the complexity introduced by structural myocardial disease and concomitant comorbidities. Likewise, Roberts et al. [5] emphasized the role of pathogenic genetic variants—particularly SCN5A mutations—as key determinants of malignant arrhythmogenic phenotypes and adverse prognostic trajectories in patients with BBR‐VT.

Our findings diverge from these prior cohorts, particularly with respect to patients without overt structural heart disease, in whom catheter ablation provided durable arrhythmia suppression and excellent survival. These observations reinforce the hypothesis that, in selected individuals with structurally normal hearts, BBR‐VT may represent a distinct clinical entity with a more indolent natural history and superior postablation prognosis. This nuanced understanding has important implications for risk stratification and therapeutic decision‐making in this population.

Patients with preserved left ventricular function were younger, exhibited shorter QRS duration during sinus rhythm, and had longer PR intervals, primarily due to a prolongation of the HV interval. An important finding is the faster rate of tachycardia observed in the group of patients with normal ventricular function. This observation may explain the occurrence of sudden death in this population, despite the presence of a structurally normal heart, as previously reported by Merino [11].

These findings expand the understanding of BBR‐VT and underscore the need for tailored diagnostic and therapeutic strategies in patients without evident structural heart disease as demonstrated in previous studies [17].

## 6 Conclusion

Although traditionally associated with patients with dilated cardiomyopathy, our study included nearly half of BBR‐VT cases in patients without structural heart disease. Once these patients are identified, Steinert's disease should be considered as a potential underlying condition. We strongly recommend ophthalmologic evaluation following electrophysiological study and radiofrequency ablation. In the absence of ocular abnormalities, genetic testing may be warranted, particularly in the attempt to identify SCN5A mutations. Right bundle branch ablation is effective in most cases; however, device implantation is often required to ensure optimal long‐term management.

## Conflicts of Interest

The authors declare no conflicts of interest.

## Data Availability

The data that support the findings of this study are available from the corresponding author upon reasonable request.
